# Identification of a new QTL underlying seminal root number in a maize-teosinte population

**DOI:** 10.3389/fpls.2023.1132017

**Published:** 2023-02-07

**Authors:** Kailiang Wang, Zhen Zhang, XiaoQian Sha, Peng Yu, Yongxiang Li, Dengfeng Zhang, Xuyang Liu, Guanhua He, Yu Li, Tianyu Wang, Jie Guo, Jiafa Chen, Chunhui Li

**Affiliations:** ^1^ College of Agriculture, Shanxi Agricultural University, Jinzhong, China; ^2^ College of Life Sciences, Henan Agricultural University, Zhengzhou, China; ^3^ Institute of Crop Sciences, Chinese Academy of Agricultural Sciences, Beijing, China; ^4^ Crop Functional Genomics, Institute of Crop Science and Resource Conservation (INRES), University of Bonn, Bonn, Germany; ^5^ Emmy Noether Group Root Functional Biology, Institute of Crop Science and Resource Conservation (INRES), University of Bonn, Bonn, Germany

**Keywords:** domestication, RNA-Seq, root development, *Zea mays*, QTL

## Abstract

Seminal roots play an important role in acquisition of water and nutrients by maize seedlings. Compared with its teosinte ancestor, maize underwent a change in seminal root number (SRN). Although several key genes controlling SRN have been cloned, identification and utilization of new genes from teosinte would be useful for improving maize root architecture. In this study, a maize-teosinte BC_2_F_6_ population containing 206 individuals genotyped by resequencing was used to conduct high-resolution quantitative trait locus (QTL) mapping of SRN. A new major QTL on chromosome 7 (*qSRN7*) was identified. Differentially expressed genes (DEGs) based on RNA-Seq were identified between two inbred lines with no SRN and multiple SRN at two periods of seminal roots primordia formation. A total of 116 DEGs detected in at least one period were identified within the *qSRN7* interval. Three DEGs (Zm00001d021572, Zm00001d021579 and Zm00001d021861) associated with SRN were identified through regional association mapping. When compared with reported domestication-related selective sweeps, Zm00001d021572 was selected during maize domestication. Our findings provide important insights into the genetic basis of SRN and identify a promising candidate gene for further studies on SRN.

## 1 Introduction

Maize (*Zea mays* ssp*. mays* L.) is one of the most important crops worldwide. It was domesticated ~9,000 years ago from Mexican lowland annual teosinte (*Zea mays* ssp. *parviglumis*). Maize has several major morphological differences from its ancestor (Matsuoka et al., 2002). In regard to aboveground morphological traits, teosinte has multiple tillers and lateral branches, small ears and few seeds with hard cupulate fruit cases, whereas maize is usually unbranched and has few tillers, but large ears with many rows of seeds that are not covered by hard cupulate fruit cases (Doebley, 2004). For underground root traits, Burton et al. (2013) documented that on average, the number of seminal roots in teosinte is about 0.5, whereas the number in cultivated maize varies from 0 to 8. Roots play crucial functions such as anchoring the plant to the ground, acquiring resources from the soil, and providing mechanical support to the stem. A root ideotype would be resilient against biotic and abiotic stress (Lynch, 2019). The maize root system is usually composed of one embryonic primary root, several seminal roots, and many crown, brace and lateral roots (Hochholdinger, 2009). The seminal root is essential for seedling survival and development during the first 2-3 weeks of seedling growth (Hochholdinger et al., 2004; Lynch, 2013). Seminal root primordia are initiated at the scutellum node about 25 days post pollination (Erdelska and Vidovencova, 1993). Previous studies reported that seminal roots penetrate the soil earlier than postembryonic roots (Weaver, 1926), and that increased seminal root number (SRN) not only contributed to total phosphorus and nitrogen uptake in the first 25 days of seedlings growth (Perkins and Lynch, 2021), but also enhanced drought tolerance in maize seedlings (Guo et al., 2020).

Variation in SRN is typically quantitative. Based on linkage mapping, previous studies identified more than 54 QTLs in different populations (Guo et al., 2018). For example, Zhu et al. (2006) used a recombinant inbred line population derived from a cross between B73 and Mo17 to detect six QTLs for SRN under high and low phosphorus conditions. Pestsova et al. (2016) identified a QTL for SRN on chromosome 8 under conditions of nitrogen deficiency in a doubled haploid line (DHL) population. Salvi et al. (2016) used an introgression library (IL) derived from landrace Gaspe as the donor parent and B73 as the recipient parent, to detect three QTLs. Wang et al. (2019) conducted a Genome-wide association study (GWAS) of SRN in 297 maize inbred lines, and identified QTLs related to SRN on chromosomes 2 and 8. Ma et al. (2021) identified a quantitative trait nucleotide (QTN) associated with SRN in an association panel containing 362 inbred lines. Although many QTLs/QTNs for SRN were identified using modern maize inbred lines, an understanding of the genetic basis of SRN and its evolution remains inadequate.

Few genes underlying SRN have been cloned following genetic analysis of mutants. The *RTCS* gene encodes a lateral organ boundaries domain (LBD) protein involving in seminal and crown root primordium formation and maintenance (Hetz et al., 1996; Taramino et al., 2007). The *RUM1* gene encodes a monocot-specific Aux/IAA protein that controls formation of seminal and lateral roots at the position of the primary root (Woll et al., 2005; von Behrens et al., 2011). The *BIGE1* gene cloned following isolation of a *bige1*-UMU5 mutant, encodes a multidrug and toxin extrusion (MATE) transporter, and regulates the timing and rate of initiation of seminal roots and plant lateral organs in both seed and plant development (Suzuki et al., 2015). Previous studies reported that elite alleles of several key genes controlling morphological traits derived from teosinte play important roles in increasing grain yield and enhancing biotic and abiotic stress (Tian et al., 2019; Wang et al., 2021; Barnes et al., 2022; Chen et al., 2022). Hence, identification of candidate genes and their alleles underlying SRN from teosinte might facilitate the use of novel genetic diversity in maize breeding.

In this study, we performed high-resolution QTL mapping for SRN, using a BC_2_F_6_ population (hereafter, TP population) derived from a cross between teosinte and inbred line PH4CV and genotyped by resequencing. Two inbred lines with large differences in SRN from an association panel were used for RNA-Seq analysis to identify DEGs related to the formation and development of SRN. Regional association mapping of SRN was carried out in an association panel containing 351 inbred lines. Combining the results from the two approaches, candidate genes underlying SRN were identified, and would provide promising genetic resources for improving maize root architecture.

## 2 Materials and methods

### 2.1 Plant materials

A TP population containing 206 individuals was derived from a single F_1_ seed from a cross between inbred line PH4CV as female parent and *Zea mays ssp*. *mexicana* (CIMMYT accession number 249743). In addition, an association panel including 351 elite inbred lines derived from a large association mapping population (Li et al., 2022) was used to conduct regional association mapping.

### 2.2 Genotyping and recombination bin map construction

Genomic DNA from individuals in the TP population was extracted by the CTAB method. Genome sequencing libraries were sequenced using the Illumina Hiseq X platform, yielding a total of 1.18 Tb raw sequences (average depth 2.5×) with 150-bp paired-end reads. After checking and filtering sequence quality, the remaining sequences were mapped to the B73 reference genome (B73_V4). A total of 62,217,878 single nucleotide polymorphisms (SNPs) were identified in the population. To obtain high-quality SNPs, we removed low-quality SNPs with missing rate >60%, minor allele frequency (MAF) > 0.175 or MAF <0.075, and heterozygosity rate >5%. Finally, 138,208 high-quality SNPs were used to construct a recombination bin map.

The R package “binmapr” was used to fix genotypes using a windows size set to 15. Redundant markers were removed by the “bin” function in IciMapping software. The genetic distances between the final 1,951 bin markers were determined using a Kosambi method in IciMapping software with the “map” function. The linkage map and recombination bin map were drawn in R software with the “LinkageMapView” package and “plot” function, respectively.

### 2.3 Phenotypic data collection and statistical analysis

To phenotype SRN, fifteen seeds of each line from the TP population and association panel were surface sterilized with 6% hypochlorite for 10 min, followed by three washes in distilled water, transferred to wet filter paper, rolled up and placed in plastic buckets containing distilled water. The buckets were incubated in darkness at 25°C. Three days later, the buckets were transferred to a new incubator at 23°C\8h darkness and 28°C\16h light. The distilled water in the buckets was replaced for every two days. After five days, the number of seminal roots for 10 healthy plants of each line was manually counted and average values of SRN were subsequently used in analysis.

### 2.4 QTL mapping for SRN

QTL analysis of SRN based on linkage map and phenotypic values from the TP population was conducted using WinQTL Cartographer v2.5 software by composite interval mapping (CIM) (Wang et al., 2012), with a 1,000 permutations test at 95% confidence level to determine the optimal logarithm of odds (LOD) threshold values. A 1.5-LOD drop method was applied for defining the QTL confidence interval. Names were assigned to QTL following the nomenclature proposed by McCouch (1997), which combines the letter “q” for QTL, an abbreviation for the name of the trait, and a number for the chromosome.

### 2.5 RNA sequencing and data analysis

Inbred lines IA2132 and PHW30 from the association panel were grown in a culture room. The number of seminal roots was determined from the seminal root primordia, which are initiated at the scutellar node of kernel about 25 days after pollination (Erdelska and Vidovencova, 1993). Samples from each inbred line for RNA-Seq were collected at the kernels scutellar node at the 20^th^ and 30^th^ days after pollination, respectively. Four biological replicates for each line were collected at each sampling time. All samples were immediately frozen in liquid nitrogen and stored at -80°C for RNA isolation. RNA extraction and library preparation for each sample were performed by Novogene Corporation. Sixteen libraries were sequenced using the Illumina HiSeq 2000 platform (Illumina, CA, USA), and 150 bp paired-end reads were generated. The raw reads were filtered to remove sequencing adapters as well as low quality reads (base number of Qphred ≤ 20 accounting for more than 50% of the read length). HISAT2 software was used to compare clean reads quickly and accurately with the reference genome to obtain the locations of reads on the AGv4 reference genome (Mortazavi et al., 2008). The FPKM (expected number of Fragments Per Kilobase of transcript per Million base pair sequences) was used to estimate gene expression levels (Trapnell et al., 2010). Differential expression analysis between the two inbred lines was performed *via* the pairwise comparison algorithm DESeq (Anders et al., 2013). DEGs were screened according to the following criteria: |log2 foldchange (FC)| ≥ 1, false discovery rate (FDR), and an adjusted P-value < 0.05 (Robinson et al., 2010). Gene Ontology (GO) enrichment analysis of the DEGs was conducted to identify the enriched biological functions between the two genotypes (Yu et al., 2012).

### 2.6 Regional association mapping underlying QTL *qSRN*7

The genotype data from the association panel of 351 inbred lines in the *qSRN7* interval were extracted from the resequencing data of 1,604 maize inbred lines reported by Li et al. (2022). After filtering using missing rates < 20%, MAF >0.05, a total of 33,164 high-quality SNPs were obtained. Principal component analysis (PCA) and kinship (*K*) matrix were calculated using 43,252 SNPs identified by the 50K SNP chip in the association panel. A mixed linear model (MLM) with PCA and *K* matrix was used to conduct regional association mapping in TASSEL 5.0 software (Bradbury et al., 2007). To determine the significant threshold for regional association results, we estimated the number of independent SNPs by pruning SNPs in the PLINK (window size 100, step size 50 and r^2^ ≥ 0.2). After pruning, the number of independent SNPs in the *qSRN7* interval was determined to be 6,070. We then selected 1.65×10^-4^ (1/6070) as the threshold of association signals.

## 3 Results

### 3.1 Construction of a recombination bin map

**Figure 1:**
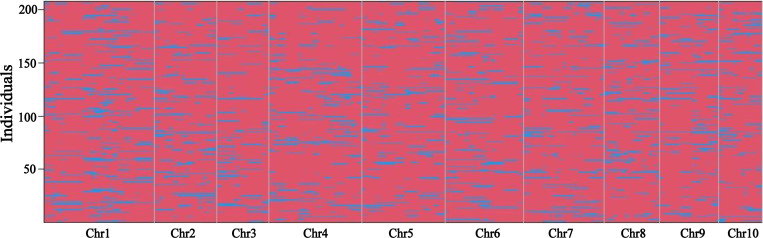
Recombination bin map of the TP population. Bin map consists of 1,951 markers. The physical position of markers is based on the B73 RefGen_v4 sequence. Red: PH4CV genotype; Blue: teosinte genotype.

The TP population was genotyped using whole genome resequencing technology. A total of 138,208 high-quality SNPs were identified in the TP population and used to determine bin markers. Finally, a total of 1,951 recombination bins were obtained by using the “binmapr” package in R software (**Figure 1**). The average physical interval of adjacent bins was 1,104.7 kb, with a maximum of 65,441.9 kb and minimum of 0.4 kb. Bin markers were mapped to maize B73 RefGen_V4 to assess the quality and accuracy of the map. The scatter plot of physical position of bin markers on all 10 chromosomes aligned well with the B73 reference genome, which indicated good collinearity between the maize B73 reference genome and the bin markers. The genetic length of the linkage map constructed using the 1,951 bin markers (**Supplementary Figure 1**) was 1,142.1 cM with an average distance of 0.59 cM between adjacent markers. The number of markers per chromosome ranged from 102 (chromosome 9) to 329 (chromosome 4), with an average of about 195 bin markers per chromosome.

### 3.2 QTL mapping for SRN

**Figure 2:**
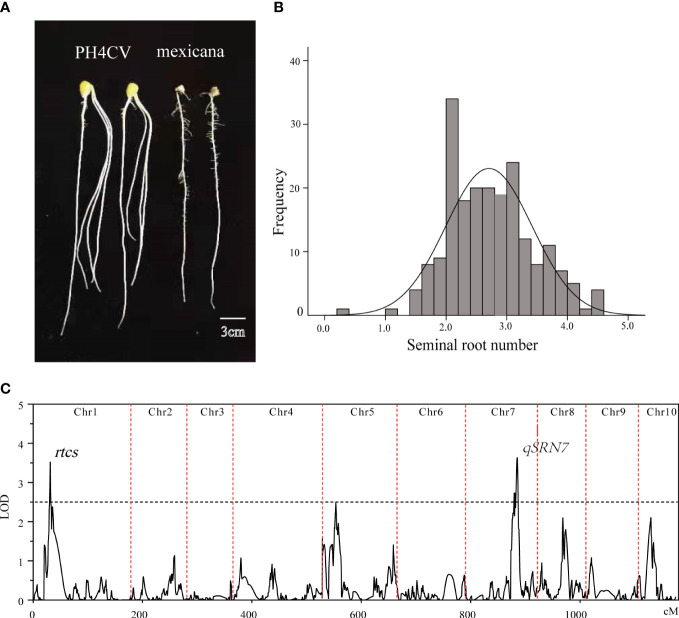
QTL mapping of SRN. **(A)** SRN in PH4CV and teosinte. **(B)** Frequency distribution of SRN in the TP population. **(C)** QTLs detected for SRN in the TP population.

We observed that the SRN showed a large phenotypic difference between the PH4CV and teosinte parents (**Figure 2A**). The average SRN of the female parent PH4CV was 3.2, whereas the teosinte parent had no seminal roots. In the TP population developed from the cross between PH4CV and teosinte, SRN ranged from 0.3 to 4.5, with a coefficient of variation (C.V.) of 26.3% (**Figure 2B** and **Supplementary Table 1**). Two QTLs were detected on chromosomes 1 and 7 (**Figure 2C**), explaining 6.18% and 6.25% of phenotypic variation, respectively. The known *RTCS* gene that has been cloned and regulates SRN, was located near the QTL peak on chromosome 1. The new QTL on chromosome 7 was named *qSRN7*, and a total of 345 candidate genes were located within the QTL interval.

### 3.3 Transcriptome sequencing analysis

**Figure 3:**
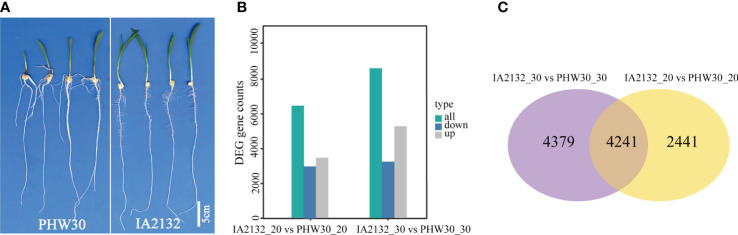
Transcriptome sequencing of two inbred lines with extreme difference in SRN. **(A)** SRN difference between PHW30 and IA2132. **(B)** DEGs between PHW30 and IA2132 at two sampling times. IA2132_20 vs PHW30_20 and IA2132_30 vs PHW30_30 represent DEGs at 20^th^ and 30^th^ days after pollination, respectively. **(C)** Venn diagram shows overlapping DEGs between IA2132_30 vs PHW30_30 and IA2132_20 vs PHW30_20.

We conducted a comparative transcriptome profiling of two inbred lines with extreme phenotypic differences in SRN. After filtration of low-quality sequences and adaptor sequences, a total of 670 million clean reads were obtained for the 16 RNA libraries. On average, 86.7% of reads were mapped uniquely to the B73 reference genome (AGPv4) (**Supplementary Table 2**). Pearson correlation coefficients among the different biological replicates of the same genotype varied from 0.92 to 0.99 (**Supplementary Figure 2A**), suggesting high quality of the replicates. In the 16 samples, 59.8% of expressed genes were expressed at low levels (0 ≤ FPKM1< 1), 37.2% were expressed at medium levels (1 ≤ FPKM < 60), and 2.96% were expressed at high levels (FPKM ≥ 60) (**Supplementary Figure 2B**). To determine which expressed genes correlated with SRN development, DEGs were analyzed between line IA2132 with no SRN and PHW30 with multiple SRN at both sampling times (**Figure 3A**). Totals of 6,682 (3,114 down-regulated and 3,568 up-regulated) and 8,620 DEGs (3,397 down-regulated and 5,223 up-regulated) genes were identified at the 20^th^ and 30^th^ days after pollination, respectively (**Figure 3B**), with 4,241 DEGs shared in both samplings (**Figure 3C**). When compared with the *qSRN7*, 116 DEGs detected at least once were located within the *qSRN7* interval (**Supplementary Table 3**).

Among the DEGs, we successfully identified *RTCS* (Zm00001d027679) and *BIGE1* (Zm00001d012883) at 30^th^ days after pollination, both genes regulate initiation of seminal roots in maize. We also identified promising DEGs associated with root development and growth. For example, *ZmEXPB2* (Zm00001d029899) encoding an expansin-B4 protein, which increases primary root length (Zainab et al., 2021), was identified at both sampling times. Zm00001d029592 encoding a root hair defective (RHD3) protein, homologous to *Arabidopsis RHD3* involved in regulating cell expansion and normal root hair development (Zheng and Chen, 2011), was also identified at .both times. GO analysis of shared DEGs identified at both sampling times revealed enrichment of genes in protein homodimerization activity, protein heterodimerization activity, heme binding, and tetrapyrrole binding (**Supplementary Figures 3A, B**). Previous studies documented that the interaction between RTCS and its paralogue RTCS-LIKE (RTCL) could form heterodimerization to regulate shoot-borne root initiation in maize (Majer et al., 2012), and RUM1 and its homolog RUM1-LIKE1 (RUL1) regulating seminal and lateral root formation can homo and heterodimerize *in vivo* (Zhang et al., 2016). These results suggested that those DEGs might be considered important candidate genes regulating seminal root formation in maize.

### 3.4 Regional association mapping underlying QTL *qSRN7*

**Figure 4:**
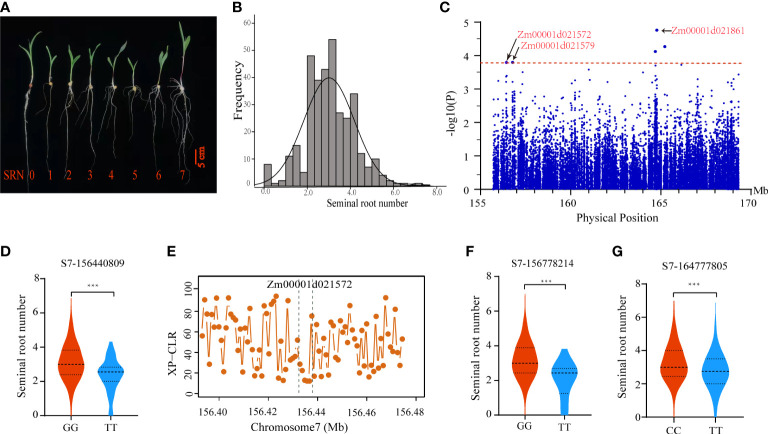
Identification of candidate genes underlying SRN. **(A)** SRN phenotypes of representative inbred lines in the association panel. **(B)** Frequency distribution of SRN in the association panel. **(C)** Manhattan plot of regional association mapping. **(D)** Phenotypic analysis of inbred lines with different haplotypes for associated SNPs (S7_156440809). **(E)** XP-CLR scores of Zm00001d021572 selected during maize domestication. The two dashed lines represent the physical position of Zm00001d021572. **(F, G)** Phenotypic analysis of inbred lines with different haplotypes for associated SNPs (S7_156778214 and S7_164777805). Center lines indicate the medians and lines above and below the median are 25^th^ and 75^th^ percentiles, and the whiskers extend 1.5 times the interquartile range from the 25^th^ and 75^th^ percentiles. Statistical significance was determined using two-sided *t*-tests. ***, *P <*0.001.

To identify candidate genes underlying *qSRN7*, we conducted regional association mapping in the association panel of 351 inbred lines. The panel showed large phenotypic differences in SRN ranging from 0 to 7 (**Figures 4A, B** and **Supplementary Table 4**). Using the MLM model in TASSEL 5.0 software, we performed an association analysis of SRN within the *qSRN7* interval. Five associated SNPs were detected at a threshold of −log10(*P*) > 3.78 (**Figure 4C**). We detected five candidate genes based on the physical location of those associated SNPs, including Zm00001d021861, Zm00001d021857, Zm00001d021572, Zm000001d021579 and Zm00001d021874.

### 3.5 Identification of high-confidence candidate genes for SRN

To identify high-confidence candidate genes underlying *qSRN7*, we integrated DEGs obtained by RNA-Seq and regional association results. Three of the 5 candidate genes obtained by regional association analysis were differentially expressed between the inbred line with no SRN and the inbred line with multiple SRN; they were Zm00001d021572, Zm00001d021579 and Zm00001d021861. For Zm00001d021572 (encoding an anthocyanidin 3-O-glucosyltransferase), an associated SNP (S7_156440809) located within the UTR3 region, could form two haplotypes. Inbred lines with the GG haplotype had significantly more seminal roots than those with TT haplotype (**Figure 4D**). Morphologically, SRN of modern maize inbred lines differ substantially from their ancestors. To ascertain whether Zm00001d021572 underwent selection during maize domestication, we searched for this gene in selected regions by comparing maize and *parviglumis* using the XP-CLR method reported by Chen et al. (2022). We found that Zm00001d021572 was selected during maize domestication (**Figure 4E**). For Zm00001d021579 (encoding a DUF869 domain-containing family protein), an associated SNP (S7_156778214) located within the exon, could form two haplotypes. Inbred lines with TT haplotype had significantly more seminal roots than those with GG haplotype (**Figure 4F**). Zm00001d021861 encodes a vacuole membrane protein KMS1, which is involved in root development in *Arabidopsis* (Wang et al., 2011). An associated SNP (S7_ 164777805) located near the physical position of Zm00001d021861, could form two haplotypes. Inbred lines with CC haplotype had significantly more seminal roots than those with TT haplotype (**Figure 4G**). The expression data of these three candidate genes (Zm00001d021572, Zm00001d021579 and Zm00001d021861) in embryo, endosperm, seed, silks, tassel, cob, leaf, internode and root tissues were downloaded from MaizeGDB. The results showed that these genes had relatively high expression in the root tissues (**Supplementary Figure 4**). These results suggested that all three candidate genes might play important roles in regulating SRN in maize.

## 4 Discussion

Maize and teosinte show large differences in morphology and environmental adaptation (Doebley, 2004). For example, teosinte has many tillers and lateral branches, small ears and fewer seeds than modern cultivated maize, and several key genes controlling these morphologic differences between teosinte and modern maize have been cloned (Clark et al., 2006; Stitzer and Ross-Ibarra, 2018). For underground traits, teosinte has no or few seminal roots compared with modern cultivated maize (Burton et al., 2013). A similar pattern was reported for wild and cultivated barley (Grando and Ceccarelli, 1995). Thus domestication appears to have increased SRN in some crop species. This may be due to the growth of teosinte seedlings requiring the seeds to provide carbohydrates and nutrients before initiation of photosynthesis, resulting in teosinte needs having insufficient seed carbohydrates to guarantee the growth of the radicle and coleoptile. For modern maize, the larger seeds might have facilitated an increase in SRN through providing additional resources for early plant development and growth (Perkins and Lynch, 2021), and improved efficiency of water and nutrient acquisition (Hufford et al., 2012).

Climate change (e.g., heat, drought, floods and disease outbreaks) is adversely affecting crop yields worldwide. To facilitate adaptation to climate change, breeders will need to produce new varieties displaying both higher yield as well as improved adaptation to different environments. Hence, it is important to use more and new genetic resources to develop stress-resilient varieties for adaptation to extreme environments. Teosinte, the closest wild relative of maize is adapted to a diverse range of environments (Hufford et al., 2012), and has diverse alleles that are absent in modern maize (Mammadov et al., 2018). In this study, a maize-teosinte TP population was constructed and genotyped by resequencing, and used to conduct QTL mapping for SRN. Two major QTLs were detected. The QTL on chromosome 1 overlapped with physical location of the cloned *RTCS* gene controlling SRN, indicating the accuracy of QTL mapping based on this TP population. By comparing the physical positions of reported QTLs for SRN, we found that the other QTL-*qSRN7* on chromosome 7 was new. The teosinte allele reduced the number of seminal roots. The TP population could be used in the future to phenotype abiotic and biotic stress and disease traits to identify elite teosinte alleles.

RNA-Seq has become an effective technology to detect expressed genes related to development and growth traits in maize, such as kernel development (Li et al., 2022) and drought tolerance (Liu et al., 2020). To our knowledge, there have been few RNA-Seq studies on seminal root primordia, which ultimately determine the number of seminal roots. We used RNA-Seq to identify DEGs underlying SRN between two inbred lines with no and multiple SRN at two sampling dates. Totals of 6,682 and 8,620 DEGs were identified at the 20^th^ and 30^th^ days after pollination, respectively, with 4,241 DEGs shared between both stages. Genes *RTCS* and *RUM1* underlying SRN were identified previously. The *RTCS* gene encodes an LBD protein involved in auxin signal transduction (Taramino et al., 2007). Expression of *RTCS* is activated by binding of auxin response factor (ARF) 34 to LBD elements in the promoter (Xu et al., 2015). Among the 4,241 DEGs obtained in this study, a group of DEGs encoding LBD was detected, including Zm00001d038197, Zm00001d043036 and Zm00001d033347. Zm00001d043036 was reported to positively regulate lateral root formation in *Arabidopsis* (Cho et al., 2019). The *RUM1* gene encodes a canonical Aux/IAA protein that is a central regulator of auxin signaling. Interaction between RUM1 and downstream ARF in the pericycle is involved in the initiation of seminal roots (von Behrens et al., 2011). In this study, many genes encoding plant root initiation and development ARF were identified among the 4,241 DEGs, such as Zm00001d031522, Zm00001d032683 and Zm00001d045026. These results provide valuable information for further studies of those DEGs and their effects on SRN.

One hundred and sixteen DEGs were detected within the *qSRN7* interval. Functional annotations indicated that many DEGs were involved in root development. For example, Zm00001d021861 encodes a vacuole membrane protein KMS1 and an *Arabi*dopsis KMS1 RNAi line exhibited shorter roots than the wild type (Wang et al., 2011). Zm00001d021745 encodes D-type cyclin, which is mainly expressed in CEI (cortex/endodermal initials) and CEID (CEI daughter) cells during early root development in *Arabidopsis* (Di Laurenzio et al., 1996; Wysocka-Diller et al., 2000; Sozzani et al., 2010; Yang et al., 2022). Zm00001d021724 encodes an F-box domain-containing protein, and overexpression of F-box protein gene *MAIF1* in rice promotes root growth and reduces abiotic stress tolerance (Yan et al., 2011). Thus, further investigation of DEGs located within the *qSRN7* interval could be helpful in identifying candidate genes underlying SRN in maize.

Previous studies suggested that regional association mapping was an effective method to identify candidate genes underlying agronomic traits. In this study, we used regional association analysis to identify SNPs significantly associated with SRN within the *qSRN7* interval (about 13.6 Mb). Five associated SNPs were identified. When combining associated SNPs and DEGs within the QTL, we identified three SNPs significantly associated with three DEGs (Zm00001d021572, Zm00001d021579 and Zm00001d021861). When genes selected during maize domestication reported by Chen et al. (2022) were compared with these three DEGs, we found that Zm00001d021572 underwent selection during domestication. The function of these three candidate genes will be validated by knockout and overexpression in future studies. Collectively, the combined use of RNA-Seq, regional association analysis and selective sweeps during domestication will be beneficial in identifying high-confidence candidate genes associated with SRN.

## 5 Conclusion

This study dissected the genetic basis of SRN in maize, through conventional QTL mapping in a maize-teosinte population genotyped by resequencing, RNA-Seq and regional association mapping. A new QTL underlying SRN was identified. A total of 4,241 DEGs shared in two sampling times were identified. Among those DEGs, we identified known genes controlling maize SRN and many candidate genes involved in root development and growth. Combining data on DEGs and associated SNPs obtained by regional association mapping within the *qSRN7* interval, three high-confidence candidate genes underlying SRN were identified. Of the three genes, Zm00001d021572 reported in a previous study was selected during maize domestication. The results of this study provide insight into the genetic basis of SRN and potential candidate genes for improving seminal root system in maize.

## Data availability statement

The data presented in the study are deposited in the Genome Sequence Archive, accession number PRJCA013887.

## Author contributions

CL, JC and JG conceived and designed the experiments. KW, ZZ and PY collected the samples to conduct resequencing and RNA-Seq. KW, ZZ and XS performed phenotypic measurement and conducted data analysis. KW, ZZ and CL wrote the manuscript. CL, JG and JC provided experimental support and participated in revising the manuscript. YXL, DZ, XL and GH participated in data analysis. TW and YL supervised the project. The authors read and approved the final manuscript.
