# Supplementary material for: Identification of a new QTL underlying seminal root number in a maize-teosinte population
Source: Front Plant Sci. 2023 Feb 7;14:1132017. doi: 10.3389/fpls.2023.1132017 (PMC9941338; doi:10.3389/fpls.2023.1132017)
Supplement: Supplementary file 1 [file DataSheet_1.pdf]

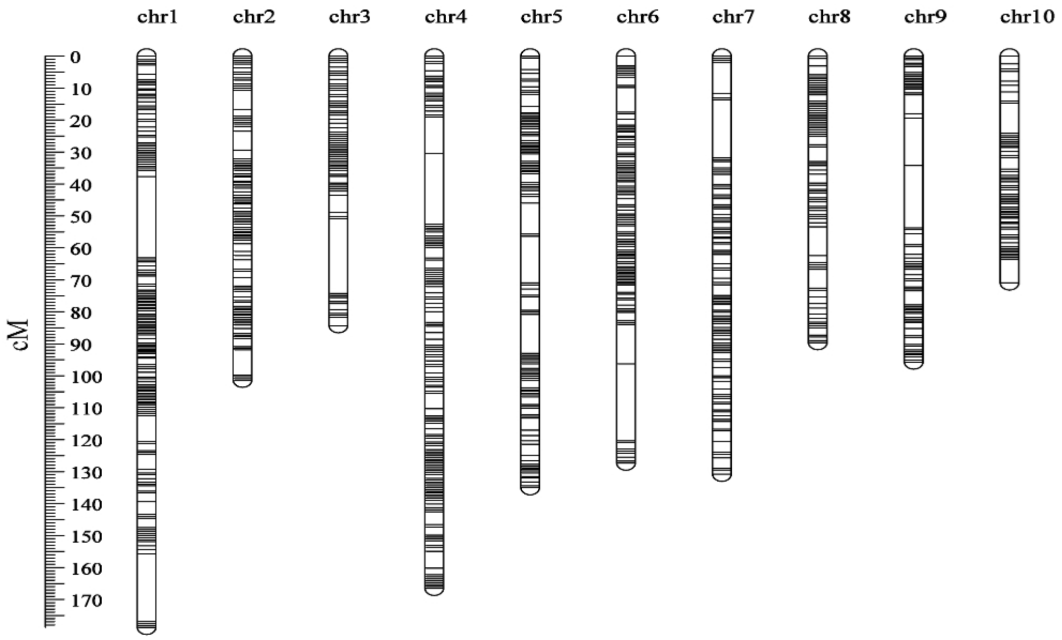

Supplementary Figure 1. The genetic linkage map constructed based on the bin makers. The black lines on each chromosome indicate the genetic location of the bin makers.
