# Supplementary material for: Identification of a new QTL underlying seminal root number in a maize-teosinte population
Source: Front Plant Sci. 2023 Feb 7;14:1132017. doi: 10.3389/fpls.2023.1132017 (PMC9941338; doi:10.3389/fpls.2023.1132017)
Supplement: Supplementary file 2 [file DataSheet_2.pdf]

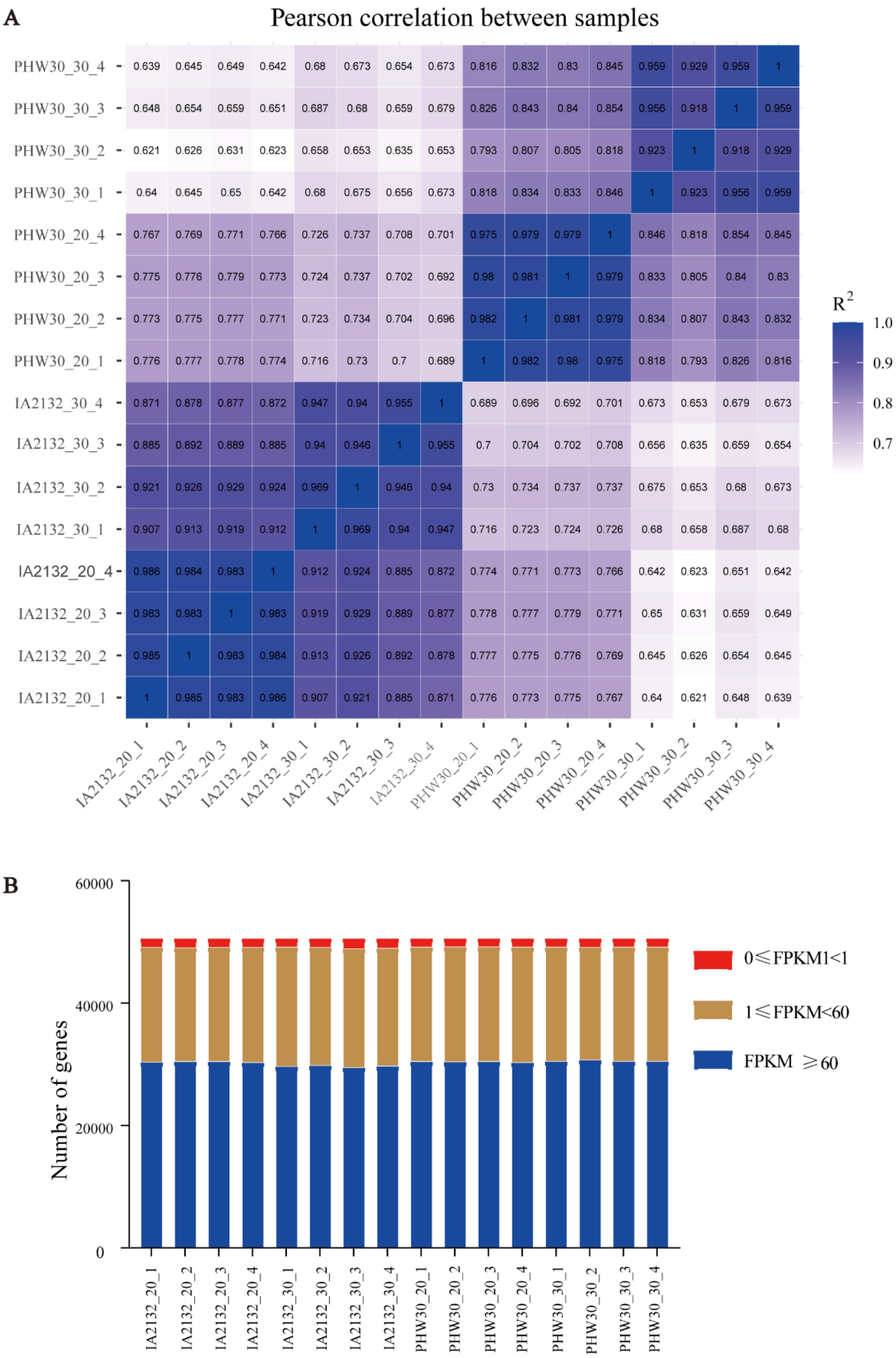

Supplementary Figure 2. Gene expression levels and their correlation among samples. (A) Heat maps showing the correlations between the transcriptomes of four biological replicates for each genotype among the two genotypes investigated at the 20th and 30th day after pollination. IA2132_20 and PHW30_20 represent DEGs at the 20th day after pollination. Similarly, IA2132_30 and PHW30_30 represent DEGs at the 30th day after pollination. (B) gene expression levels for 16 samples at the 20th and 30th day after pollination.
