# Supplementary material for: Identification of a new QTL underlying seminal root number in a maize-teosinte population
Source: Front Plant Sci. 2023 Feb 7;14:1132017. doi: 10.3389/fpls.2023.1132017 (PMC9941338; doi:10.3389/fpls.2023.1132017)
Supplement: Supplementary file 3 [file DataSheet_3.pdf]

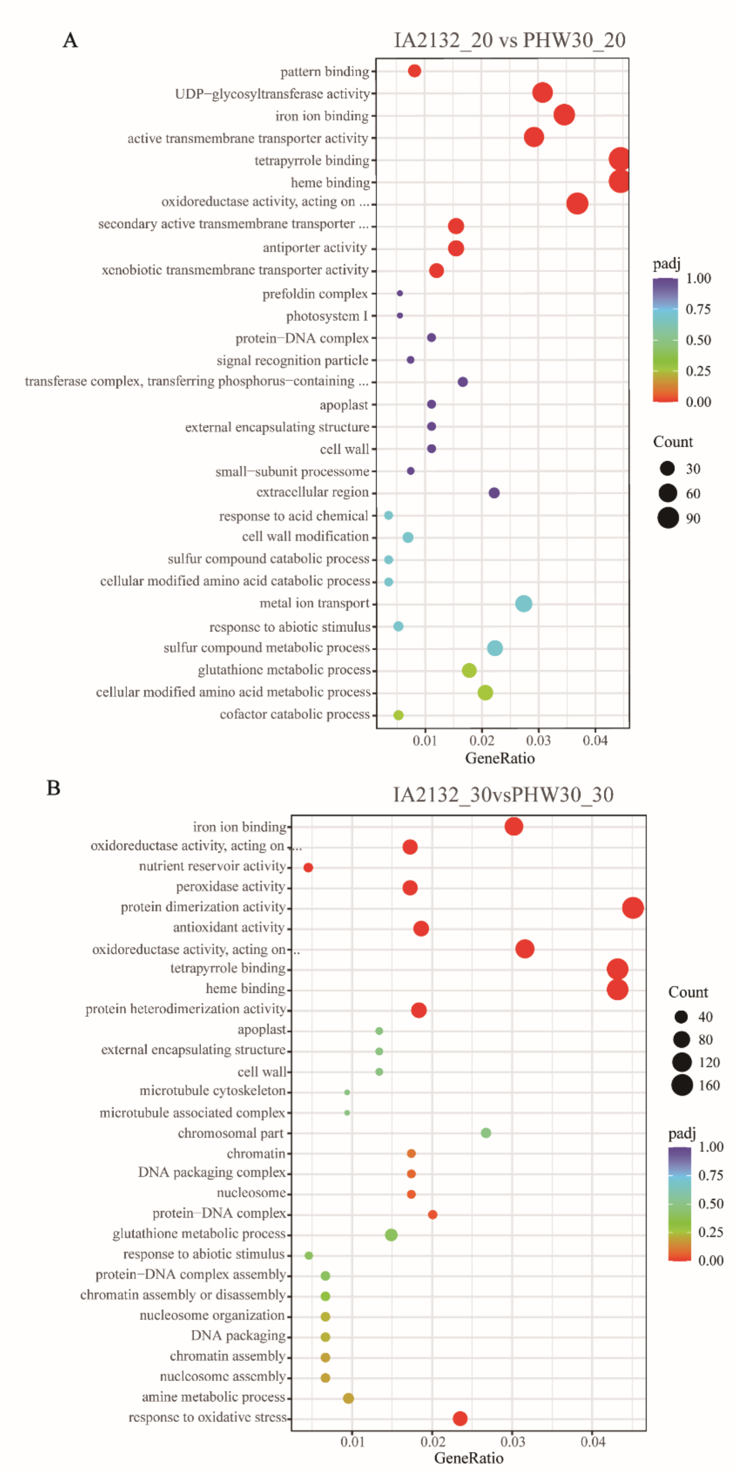

Supplementary Figure 3. GO analysis. (A) Enriched GO terms at the 20th day after pollination. In the figure, the abscissa is the ratio of the number of differentially annotated genes on GO Term to the total number of differentially annotated genes on GO Term; the vertical axis is GO Term (first 30); the size of dots represents the number of genes annotated on GO term; the color from red to purple represents the significance of enrichment, the same as below. (B) GO terms at the 30th day after pollination.
