# Supplementary figures and images for: Identification of a new QTL underlying seminal root number in a maize-teosinte population
Source: Front Plant Sci. 2023 Feb 7;14:1132017. doi: 10.3389/fpls.2023.1132017 (PMC9941338; doi:10.3389/fpls.2023.1132017)

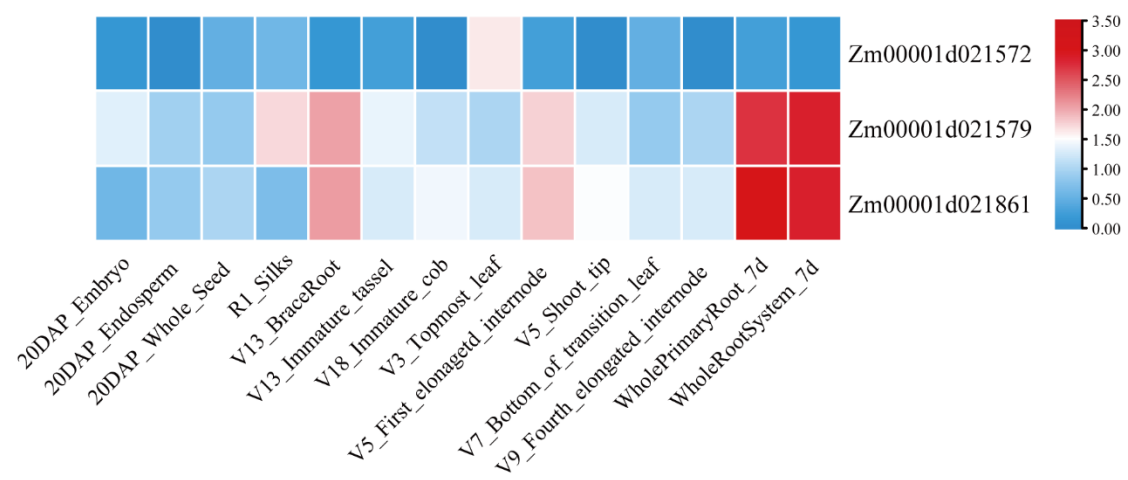

**Supplementary Figure 4.** Tissue expression heatmap of genes Zm00001d021572, Zm00001d021579 and Zm00001d021861 in maize inbred line B73.

Supplement: Supplementary file 4 [file DataSheet_4.pdf]
