# Supplementary material for: Identification of a new QTL underlying seminal root number in a maize-teosinte population
Source: Front Plant Sci. 2023 Feb 7;14:1132017. doi: 10.3389/fpls.2023.1132017 (PMC9941338; doi:10.3389/fpls.2023.1132017)
Supplement: Supplementary file 5 [file Table_1.pdf]

Supplementary Table 1. Phenotype description statistics of TP population

Number	Min	Max	Mean	SD	variance	Skewness		Kurtosis		*C. V.*
						Statistics	SEM	Statistics	SEM	
207	0.33	4.50	2.71	0.713	0.508	0.203	0.169	0.036	0.337	26.34%
