# Supplementary material for: Identification of a new QTL underlying seminal root number in a maize-teosinte population
Source: Front Plant Sci. 2023 Feb 7;14:1132017. doi: 10.3389/fpls.2023.1132017 (PMC9941338; doi:10.3389/fpls.2023.1132017)
Supplement: Supplementary file 8 [file Table_4.pdf]

Supplementary Table 4. Phenotypic description statistics of 351 maize inbred lines

Number	Min	Max	Mean	SD	variance	Skewness		Kurtosis		*C. V.*
						Statistics	SEM	Statistics	SEM	
351	0.00	7.00	3.01	1.171	1.371	0.182	0.130	0.834	0.260	38.88%
